# Hybrid Neurofibroma-Schwannoma

**DOI:** 10.7759/cureus.548

**Published:** 2016-03-30

**Authors:** Namath S Hussain, Charles S Specht, Elizabeth Frauenhoffer, Michael Glantz, Kimberly Harbaugh

**Affiliations:** 1 Department of Neurosurgery, Penn State Hershey Medical Center; 2 Pathology, Penn State Hershey Medical Center

**Keywords:** neurofibroma, schwannoma, hybrid tumor

## Abstract

Neurofibromas and schwannomas are common lesions that may be idiopathic or may occur in association with neural crest genetic syndromes such as neurofibromatosis type 1, neurofibromatosis type 2, and schwannomatosis. A hybrid tumor that contains pathological characteristics of both neurofibroma and schwannoma has been described as a rare entity. We present the clinical, radiographic, and pathological findings of such a case.

## Introduction

Most peripheral nerve tumors are benign lesions, which commonly present as a soft tissue mass, with pain, or with focal neurologic deficits, in that order of frequency [1-2]. Mast-cell mediated inflammation has been reported and likely explains the pain relief seen with antihistamine therapy in some patients. Surgical excision is often performed in medically intractable cases. Histopathological diagnostic classification schemes for peripheral nerve tumors have been published [1-2]. However, a hybrid lesion that does not fit neatly into this system has been reported; these tumors have pathological characteristics of both neurofibroma and schwannoma [3-12]. We describe a case of a 47-year-old male with multiple painful benign peripheral nerve tumors that had histopathologic characteristics of both neurofibroma and schwannoma. Informed consent was obtained from the patient for this study.

## Case presentation

**Figure 1:**
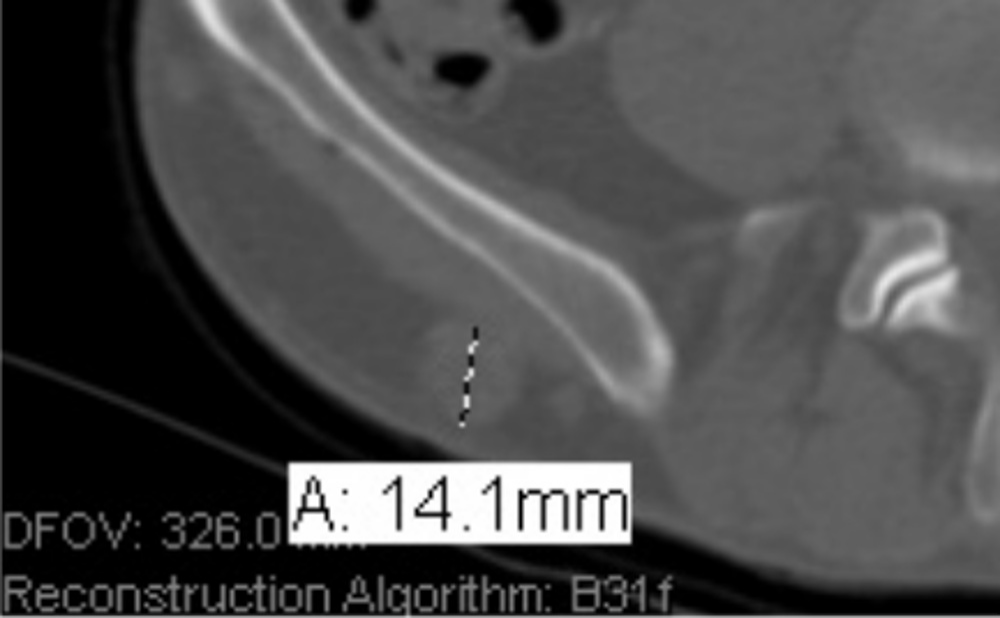
CT scan of the abdomen revealing deep tissue lesions concerning for neurofibroma.

The patient is a 47-year-old male with a history of multiple painful enlarging right-sided nerve sheath tumors on his hip, pubic region, paraspinal, and spinal foraminal regions. A formal diagnosis of neurofibromatosis was entertained but could not be made clinically based on published criteria [1]. At clinical examination, the patient lacked other diagnostic features consistent with neurofibromatosis type 1 or neurofibromatosis type 2, and genetic testing did not reveal constitutional chromosomal abnormalities diagnostic for these disorders. Segmental neurofibromatosis type 1 or neurofibromatosis type 2 were considered as the lesions were found only on the right side of the body. The patient had an extensive surgical history with more than 90 of these tumors removed. Computed tomography (CT) scans of chest, abdomen, and pelvis demonstrated multiple cutaneous and subcostal lesions (Figure 1).

**Figure 2:**
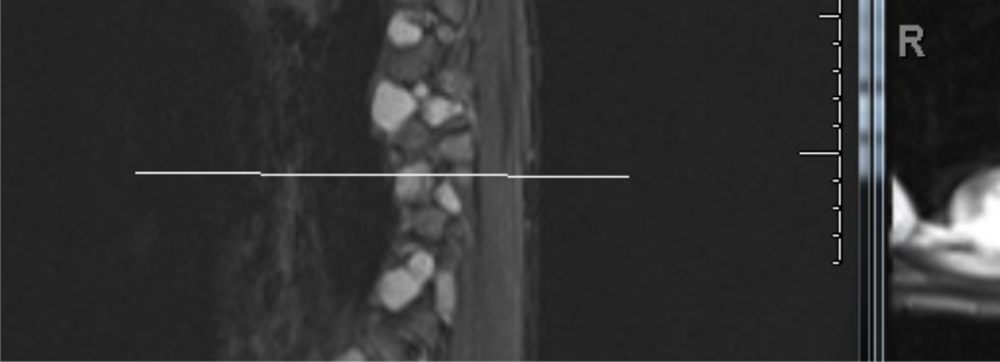
MRI of the thoracic spine with multilevel right-sided foraminal lesions.

A magnetic resonance imaging (MRI) of the brain was unremarkable. An MRI of the spine showed heterogenously enhancing right-sided masses at multiple thoracic and lumbar nerve roots (Figure 2).

These lesions were painful, and the patient’s discomfort could be temporarily ameliorated in a given area by surgical excision of a lesion until there was growth of a new lesion in the same locale. Ultimately, however, the implantation of an intrathecal drug delivery pump was required to maintain analgesia after nonsteroidal anti-inflammatory drugs (NSAIDs) were found to be ineffective. In this report we describe the pathological features of subcutaneous tumors excised from the right iliac crest, right paraspinal, and right pubic regions.

**Figure 3:**
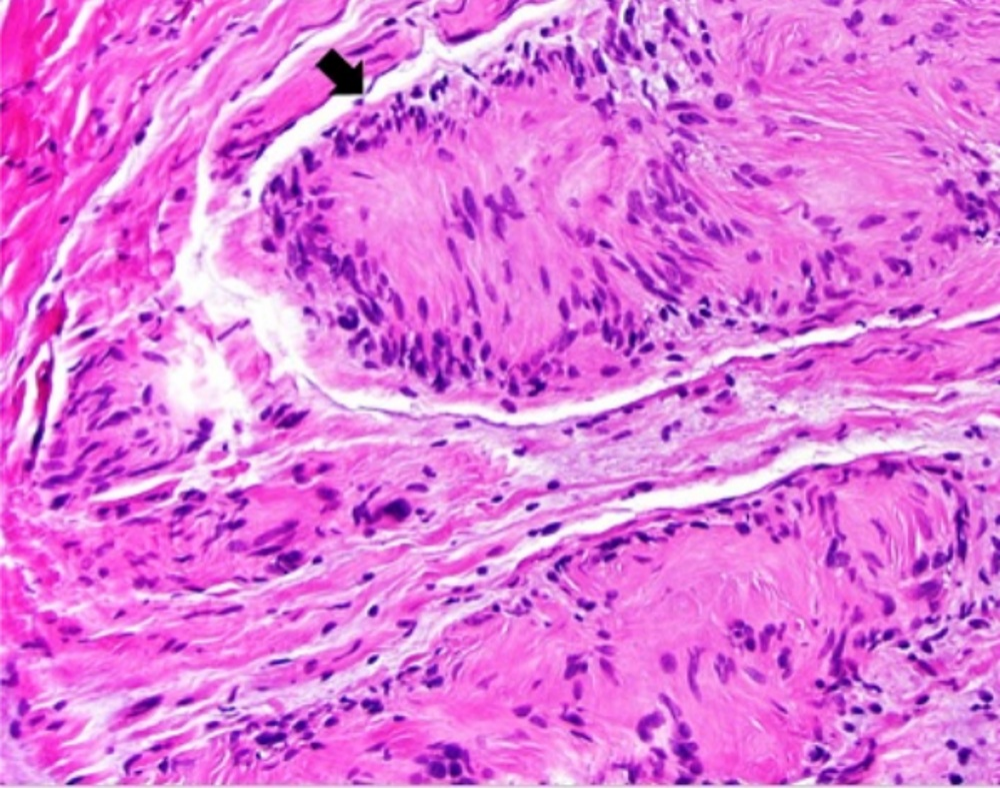
H&E, 200x. Histopathologic features of neurofibroma-schwannoma hybrid. This field illustrates mixture of plexiform neurofibroma and focal Schwann cell proliferation (arrow).

**Figure 4:**
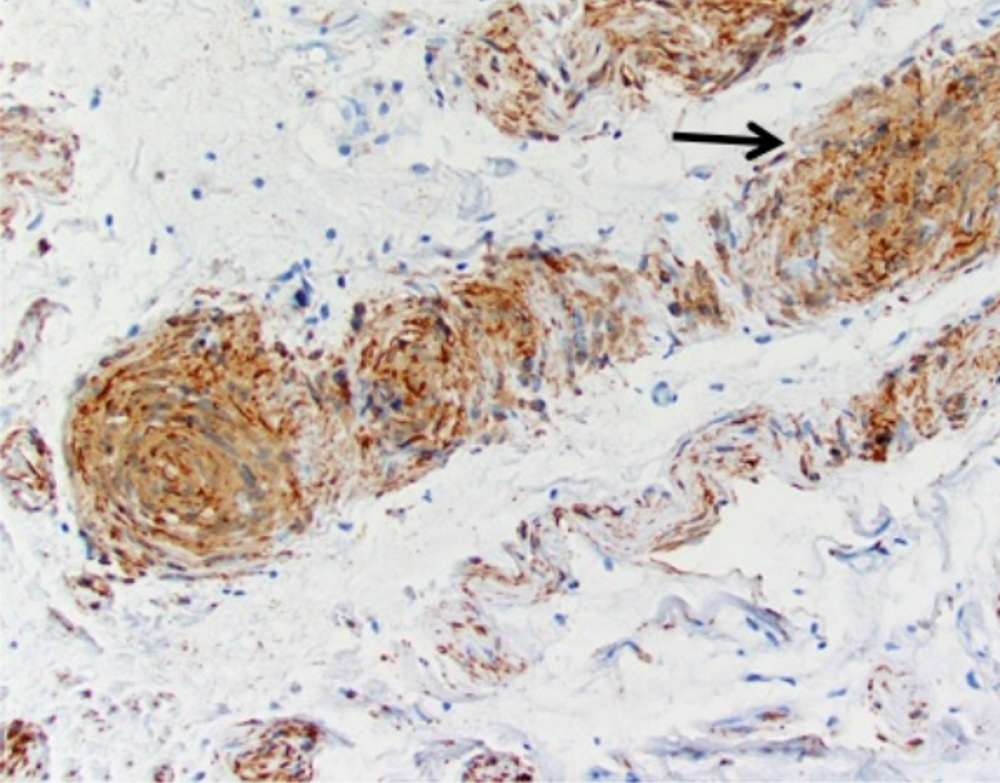
S100, 200x. Foci of Schwann cell proliferation are strongly S100-positive (arrow).

**Figure 5:**
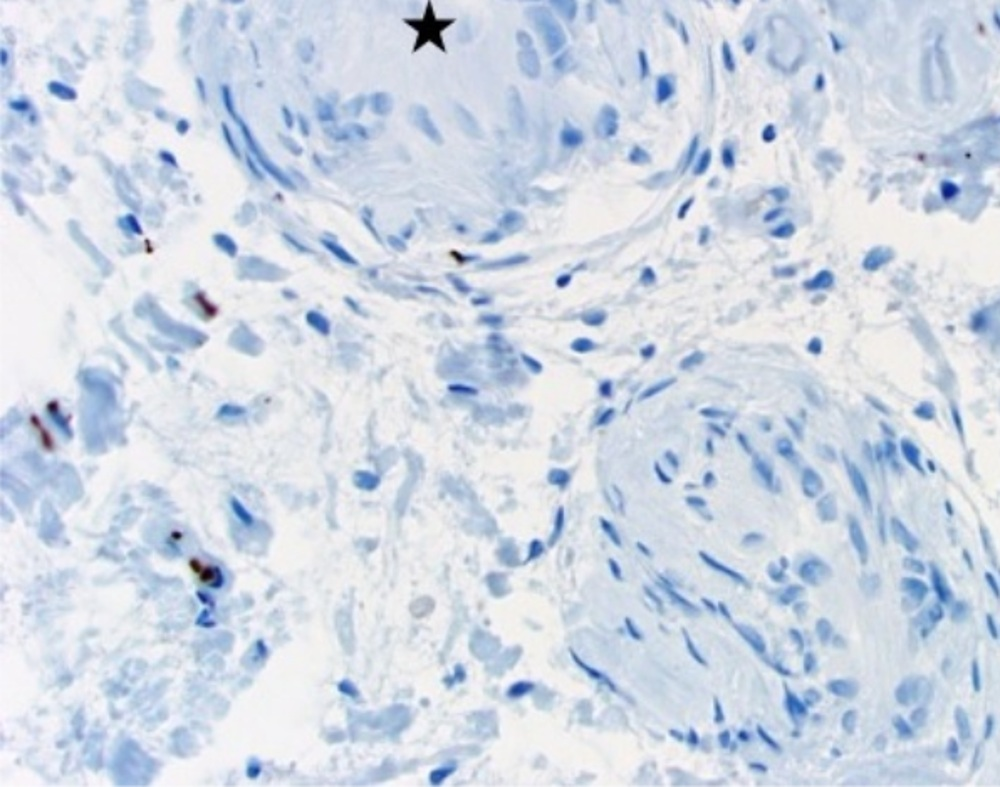
NFP, 400x. In this field, neurofilament-positive axons from the neurofibroma lie adjacent to focal Schwann cell proliferation (star).

**Figure 6:**
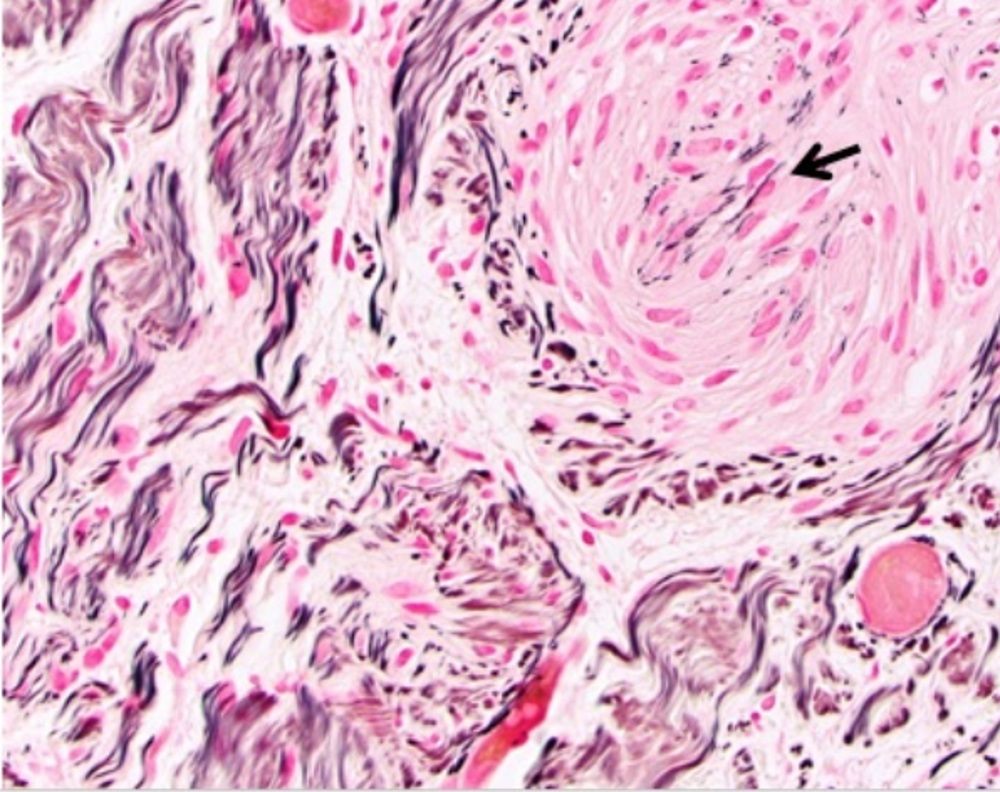
Reticulin, 400x. Note pericellular reticulin fibers in focus of Schwann cell proliferation (arrow). This highlights the basement membrane of the Schwann cell.

**Figure 7:**
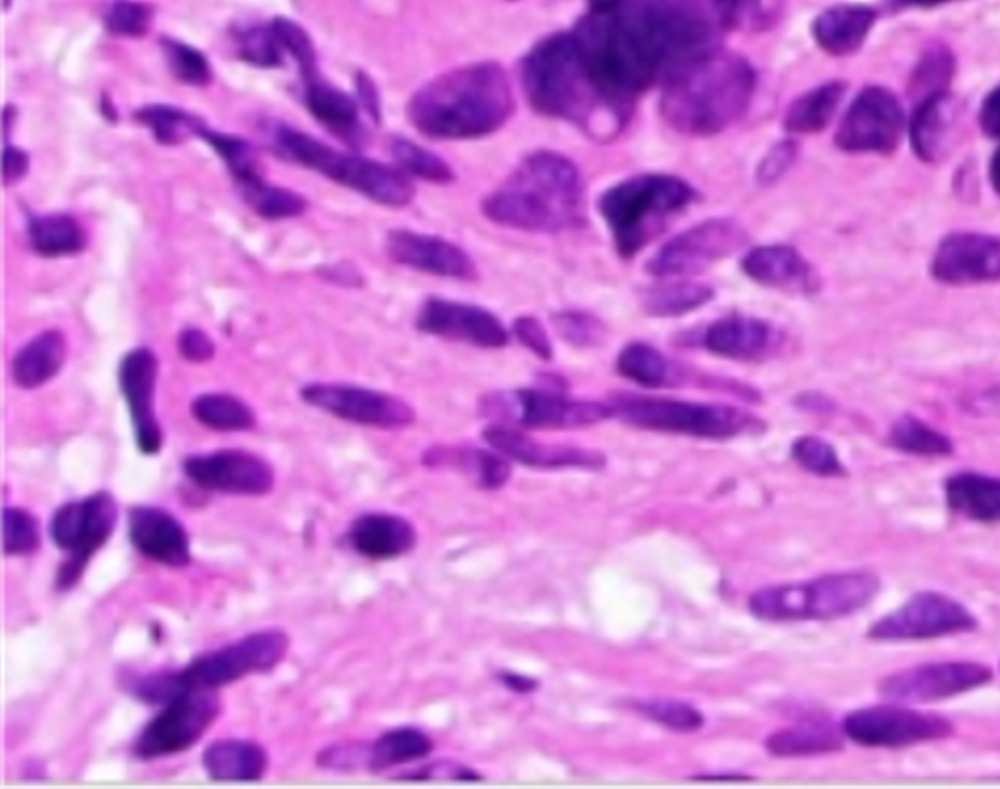
H&E, 400x. Focal atypia with hyperchromatic, mildly enlarged nuclei. See adjacent area of bland Schwann cell proliferation.

The tumors excised from the right iliac crest, right paraspinal, and right pubic regions showed similar pathologic features. On gross examination, the lesions appeared as tan-pink soft tissue nodules that ranged from 0.7 to 1.5 cm in diameter. On microscopic examination, features of plexiform neurofibroma predominated; however, there were focal regions of Schwann cell proliferation that resembled schwannoma (Figures 3-7).

Based on these findings, the lesions were considered to be hybrid neurofibroma-schwannomas. Some microscopic foci of cellular atypia were seen, but mitoses were not identified and the degree of atypia in these small superficial lesions was not sufficient to warrant a diagnosis of malignancy.

## Discussion

Traditional classification schemes label neurofibromas and schwannomas as distinct clinicopathological entities rather than lesions that exist along a spectrum [1,9]. The existence of hybrid neurofibroma-schwannoma lesions indicates that current classifications may not capture all cases and may not properly describe the true nature of the underlying pathology. The largest series of this mixed hybrid tumor was reported by Feany et al. [3] Of the nine patients they report, only one had a definite diagnosis of neurofibromatosis type 1. 

Neurofibromatosis type 1, the most common syndrome associated with nerve tumors, has a well-defined pathogenesis. Germline mutations in the NF1 gene on chromosome 17 cause this syndrome. The NF1 gene product is neurofibromin, a protein that inhibits the ras oncogene. Neurofibromatosis type 1 may be diagnosed clinically in a patient who has at least two of seven diagnostic criteria, which include café-au-lait spots, axillary freckling, Lisch nodules, neurofibromas, optic gliomas, skeletal dysplasia, and a first-degree relative with the disease [1]. There have been reports of segmental neurofibromatosis type 1 resulting from mosaicism for mutation in the NF1 gene [2,12].

Neurofibromatosis type 2 is associated with the development of multiple schwannomas; meningiomas and ependymomas may also occur. This disease is linked to mutation of the NF2 gene on chromosome 22. The NF2 gene product is merlin, a protein that regulates contact-dependent inhibition of proliferation and thus supports the formation of neoplasia. The clinical finding of bilateral vestibular schwannomas is a pathognomonic feature of neurofibromatosis type 2. After neurofibromatosis types 1 and 2 have been clinically excluded, a diagnosis of schwannomatosis may be considered. Schwannomatosis can be diagnosed in a patient who has multiple cutaneous or spinal schwannomas, but lacks vestibular schwannomas and does not exhibit clinical criteria diagnostic for neurofibromatosis type 1. These schwannomas are often painful. Most cases of well-defined schwannomatosis are sporadic and presentation as a familial syndrome is uncommon. Schwannomatosis has been linked to somatic mutation of the NF2 gene, as well as to mutations of the SMARCB1 (INI1) and the LZTR1 genes [4-6,13]. The SMARCB1 and LZTR1 gene products are proteins that regulate chromatin conformation and remodeling. The NF2, SMARCB1 and LZTR1 gene loci lie near to each other on chromosome 22, and mutations in SMARCB1 or LZTR1 may lead to conditions that are favorable to somatic alteration of NF2 gene function [14-15].

Our patient has multiple painful cutaneous and spinal nerve sheath tumors. He lacks clinical and constitutional genetic features diagnostic for neurofibromatosis type 1 or 2. With these findings, a diagnosis of schwannomatosis has been made. The patient has right-sided painful tumors that on pathological examination exhibit hybrid features of both neurofibromas and schwannomas. Such tumors have been identified in patients with schwannomatosis. The unilateral hemibody disease pattern represents a segmental presentation of schwannomatosis. This phenomenon has been recognized in a minority of reported cases.

## Conclusions

Reported clinical and pathological descriptions of hybrid neurofibroma-schwannoma are concordant with our diagnosis. Surgical excision is the treatment of choice for tumors of schwannomatosis. Focal cellular atypia was present in our patient’s resected tumors. This finding may represent the focal development of degenerative changes in schwannomatous cells, and in this case was not considered sufficient to render a diagnosis of malignancy. However, although quite rare, development of malignant peripheral nerve sheath tumor has been reported in patients with a history of schwannomatosis [16]. Clinical follow-up is important in the management of these patients.
